# Engineering of fast-growing Vibrio natriegens for biosynthesis of poly(3-hydroxybutyrate-co-lactate)

**DOI:** 10.1186/s40643-024-00801-4

**Published:** 2024-09-09

**Authors:** Xinye Sun, Yanzhe Shang, Binghao Zhang, Pengye Guo, Yuanchan Luo, Hui Wu

**Affiliations:** 1 State Key Laboratory of Bioreactor Engineering, Shanghai Frontiers Science Center of Optogenetic Techniques for Cell Metabolism, School of Biotechnology, East China University of Science and Technology, 130 Meilong Road, Shanghai, 200237 China; 2 MOE Key Laboratory of Bio-Intelligent Manufacturing, School of Bioengineering, Dalian University of Technology, Dalian, China; 3 Shanghai Collaborative Innovation Center for Biomanufacturing Technology, 130 Meilong Road, Shanghai, 200237 China; 4 Key Laboratory of Bio-based Material Engineering of China, National Light Industry Council, 130 Meilong Road, Shanghai, 200237 China

**Keywords:** Poly(3-hydroxybutyrate-co-lactate), *Vibrio natriegens*, Metabolic engineering, Lactate fraction

## Abstract

Poly(3-hydroxybutyrate-co-lactate) [P(3HB-co-LA)] is a highly promising valuable biodegradable material with good biocompatibility and degradability. *Vibrio natriegens*, owing to its fast-growth, wide substrate spectrum characteristics, was selected to produce P(3HB-co-LA). Herein, the crucial role of acetyltransferase PN96-18060 for PHB synthesis in *V. natriegens* was identified. Heterologous pathway of P(3HB-co-LA) was introduced into *V. natriegens* successfully, in addition, overexpression of the *dldh* gene led to 1.84 fold enhancement of the lactate content in P(3HB-co-LA). Finally, the production of P(3HB-co-LA) was characterized under different carbon sources. The lactate fraction in P(3HB-co-LA) was increased to 28.3 mol% by the modification, about 1.84 times of that of the control. This is the first successful case of producing the P(3HB-co-LA) in *V. natriegens*. Collectively, this study showed that *V. natriegens* is an attractive host organism for producing P(3HB-co-LA) and has great potential to produce other co-polymers.

**Supplementary Information:**

The online version contains supplementary material available at 10.1186/s40643-024-00801-4.

## Introduction

Over the past few decades, the plastic manufacturing industry has experienced a rapid growth. From 2000 to 2020, global demand for plastics nearly doubled, and it is projected to continue increasing in the future due to the expanding market of plastics (Kwon et al. 2023). However, plastic pollution has become one of the most serious environmental problems due to its adverse impact on ecosystems (Langsdorf et al. 2021; Sirohi et al. 2020). Polyhydroxyalkanoates (PHA) are considered “bio-plastics” that can be produced through renewable resources and be biodegraded by microorganisms (Dan et al. 2023; Lu et al. 2020). It became one of the ideal materials for the plastic industry. Among various PHA bio-based plastics, P(3HB-co-LA) not only exhibits excellent transparency similar to polylactic acid (PLA) (Cao et al. 2024), but also possesses the impact resistance and heat resistance of Polyhydroxybutyrate (PHB). PLA has good mechanical strength and transparency, however, P(3HB) is a fragile and opaque polymer. The copolymer of P(3HB-co-LA) combines the two characteristics and the elongation rate can be improved, thus obtaining a wider range of applications (Ali et al. 2024). By combining the advantages of these two traditional materials, P(3HB-co-LA) has improved the properties of the material and shows broad prospects in various application fields (McAdam et al. 2020).

Currently, the *de novo* synthesis of P(3HB-co-LA) has been successfully achieved by modifying microorganisms. The biosynthesis of P(3HB-co-LA) has been developed through the polymerization of two precursors 3-hydroxybutyryl-CoA (3HB-CoA) and lactyl-CoA catalyzed by *Pseudomonas fluorescens* 2P24-derived PHA synthetase mutant (PhaC1STQK) (Taguchi et al. 2008). Among them, 3HB-CoA is catalyzed from acetyl-CoA via acetyl-CoA acetyltransferase and NADPH-dependent acetylacetyl-CoA reductase, while lactyl-CoA is synthesized by propionyl-CoA transferase mutant (Pct*) from lactate as a precursor (Ta et al. 2023). Many researchers have made efforts in metabolic engineering and fermentation optimization to regulate the LA fraction, thereby expanding its application range (Guo et al. 2021). Specifically, reducing or inhibiting the consumption of pyruvate, the LA fraction in the polymer can be elevated to 70.0 mol% (Jung et al. 2010). Furthermore, altering carbon sources and cultivation conditions during fermentation can also elevate the lactate (LA) fraction. When sucrose is used as a substrate, combined with the introduction of the exogenous sucrose utilization pathway, engineered *Escherichia coli* can produce 29.4 wt% P(3HB-co-LA), accompanied by a 42.3 mol% LA fraction. Moreover, under anaerobic conditions, the LA fraction can increase by up to 8-fold (Wu et al. 2021). Meanwhile, the synthesis of P(3HB-co-LA) was also achieved by engineering different microorganisms. By modifying *Sinorhizobium meliloti*, it was enabled to utilize Yeast Mannitol medium to produce 15.0 wt% of P(3HB-co-LA) and LA fraction was increased to 30.0 mol% (Tran and Charles 2016). When *Corynebacterium glutamicum* is used as the host, it is possible to obtain more than 90.0 mol% of LA fraction polymer, and less than 2.0 wt% P (3HB-co-LA) can be accumulated (Song et al. 2012). So far, P(3HB-co-LA) has not been commercially produced, and researchers are still working to reduce the cost of synthesis and improve the product performance. Advances in metabolic engineering, such as developing new chassis bacteria, specifically modifying relevant enzymes, optimizing fermentation conditions, and utilizing more affordable carbon sources, may facilitate the timely commercialization of P(3HB-co-LA) (Shi et al. 2022).

Selecting suitable chassis strains is the primary consideration in synthetic biology, and the ideal chassis strains should exhibit excellent characteristics, such as a high growth rate, a broad substrate spectrum, simple culture conditions, and complete genomics and gene regulation tools (Gao et al. 2022). Despite their widespread use, common chassis bacteria like *E. coli* and *Saccharomyces cerevisiae* also have limitations in various aspects. Consequently, developing new chassis bacteria has become a prominent research direction in recent years. Nowadays, *V. natriegens*, a novel chassis organism strain, hailed as the “next-generation chassis organism” in synthetic biology (Thompson et al. 2020; Zhang et al. 2021a). Meanwhile, *V. natriegens* is a non-pathogenic bacterium well-suited for synthesizing food and medical material substrates such as P(3HB-co-LA). Additionally, as a natural producer of PHB, *V. natriegens* benefits from its metabolic pathways and endogenous enzymes offering distinct advantages for the production of P(3HB-co-LA). *V. natriegens* possesses capabilities of broad substrate utilization, rapid growth, fast protein synthesis and biomass production (Weinstock et al. 2016; Zhou et al. 2024). Notably, *V. natriegens* has the shortest doubling time among known bacteria, with growth rates 1.4–3.9 times faster than *E. coli* (Lee et al. 2016). This rapid growth result in higher rates of protein synthesis and enzyme activity. Consequently, *V. natriegens* can significantly accelerate the laboratory processes in synthetic biology by reducing culture times (Hoff et al. 2020). Due to these properties, *V. natriegens* serves as an exceptional host for various valuable products, including melanin, PHB, lycopene, 1,3-propanediol, L-alanine, and citramalate (Lee et al. 2024; Zhang et al. 2021b). In this work, *V. natriegens* has been chosen to produce P(3HB-co-LA). Here, key enzymes involved in the synthesis pathway of 3HB-CoA were firstly screened and identified. Subsequently, exogenous genes of the PHB biosynthesis pathway were introduced in *V. natriegens* to construct its synthesis pathway. In order to improve the accumulation efficiency of P(3HB-co-LA), the synthesis pathway of PHB was blocked by disrupting the *PN96-18060* gene. Then, *de novo* synthesis of P(3HB-co-LA) in *V. natriegens* was achieved for the first time by introducing an exogenous lactate component production module. A major influencing factor on the performance of P(3HB-co-LA) is the lactate content (Wu et al. 2021). In this study, by overexpressing lactate dehydrogenase, the intracellular accumulation of lactate was successfully increased, effectively increasing the proportion of lactate components in P(3HB-co-LA) produced by sodium dependent *V. natriegens*. Finally, considering that *V. natriegens* can utilize multiple sources, the production efficiency of P(3HB-co-LA) was tested under different carbon sources. It is the first time to synthesize P(3HB-co-LA) in engineered *V. natriegens*. This study demonstrates the enormous potential of utilizing engineered *V. natriegens* as a new platform for PHA biosynthesis, and providing strategies for the synthesis of other copolymers.

## Materials and methods

### Strains and plasmids

Wild-type *V. natriegens* (ATCC 14048) was used as the starting host for pathway engineering in this study. The plasmids pTargetF + Tfox, pTrc99a, and pBAD33 were used for gene knockout, and expression. *E. coli* DH5α was used for plasmid construction and proliferation. All of the strains and plasmids are summarized in Table S1.

### DNA manipulations and strains construction

The exogenous genes pct^*^(mutant pct) from *Clostridium propionicum* DSM 1682, *phaA* and *phaB* from *Ralstonia eutropha*, and (mutant phaC) phaC^*^ from *Pseudomonas fluorescens* strain 2P24 were codon optimized by GenScript (Nanjing, China). The overexpression of endogenous genes were amplified from the genome of *V. natriegens*. Afterward, the purified fragments were inserted into the target plasmid, followed by transformation according to the methods described in the previous study (Wu et al. 2021). The *phaC* gene from *P. fluorescens* strain 2P24 was subjected to mutagenesis (E130D, S325T, Q481K) and codon optimization to obtain phaC*. Subsequently, phaC* along with the *phaA* and *phaB* genes from *Ralstonia eutropha*, which had undergone codon optimization, were inserted into pTrc99a, resulting in pTrc99a-phaABC*. Combined with codon-optimized phaC*, *PN96-18050* and *PN96-18045* gene from *V. natriegens*, instead of *phaA* and *phaB* gene from *R. eutropha*, were inserted into pTrc99a to form pTrc99a-18050-18045-phaC*. Combined with codon-optimized phaC*, *PN96-19050* and *PN96-18045* gene from *V. natriegens*, *instead of phaA* and *phaB* gene, were inserted into pTrc99a to form pTrc99a-19050-18045-phaC*. Combined with codon-optimized phaC*, *PN96-21465* and *PN96-18045* gene from *V. natriegens*, instead of *phaA* and *phaB* gene from *R. eutropha*, were inserted into pTrc99a to form pTrc99a- 21465-18045-phaC*. The codon-optimized *pct* gene from *C. propionicum* DSM 1682 was inserted into pBAD33 to obtain pBAD33-pct*. On this basis, *dldh* was constructed in pBAD33-pct* to obtain pBAD33-pct*-dldh.

### Culture Media and conditions

*E. coli* DH5α for plasmid construction were cultured as previously described (Wu et al. 2021). *V. natriegens* were cultivated in tubes containing LB3 medium (LB broth supplemented with an additional 20 g/L NaCl) for strain construction. For the fermentation experiments, *V. natriegens* were cultured in M9NA medium (M9 medium with an additional 1.5 g/L NaCl and 5 g/L yeast extract). The formula of M9 medium consisted of the following components (per liter): 15.12 g Na_2_HPO_4_·12H_2_O, 0.5 g KH_2_PO_4_, 3.0 g, NaCl, 0.5 g MgSO_4_·7H_2_O, 0.011 g CaCl_2_, 1.0 g NH_4_Cl, 0.2 mL 1% (w/v) vitamin B1, and 0.1 mL trace elements solution. The concentration of different carbon sources, including glucose, mannitol, and sucrose, in different fermentation experiments was 10 g/L. During the cultivation process, 100 mg/L of ampicillin and/or 34 mg/L of chloramphenicol are added as needed. The seed culture for the fermentation was obtained by selecting a newly grown single colony from an agar plate and inoculating it into 5 mL of fresh LB3 medium. After overnight cultivation in a shaker (Shanghai Zhichu Instrument Co., Ltd) at 30℃ and 220 rpm, inoculate the seed culture into 50 mL (1% v/v) fermentation media in a 250 mL shake flask. Subsequently, fermentation was conducted for 36–48 h at 30 °C and 220 rpm. Additionally, add 0.1 mM IPTG to the culture medium at the beginning of fermentation to induce enzyme expression. And add appropriate antibiotics (100 mg/L ampicillin and/or 12.5 mg/L chloramphenicol) as needed to the culture medium. Samples of the fermentation were taken every 12 h for testing. During the process of fermentation, 3 M H_2_SO_4_ and 6 M NaOH were used to adjust the pH of the fermentation broth at 7-7.5. All experiments were conducted in triplicate to ensure reliable results.

### Analytical methods

The analysis method for cell growth, substrate (glucose, mannitol) and metabolite (acetate, lactate) content during the fermentation process is as described in previous study (Wu et al. 2021). The monomer content of P(3HB-co-LA) was also determined using the previously described method (Wu et al. 2021). The detection conditions for sucrose employed a sucrose detection kit obtained from Nanjing Jiancheng Institute of Biotechnology.

## Results and discussion

### Identification of key enzymes in the synthesis pathway of the precursor 3-hydroxybutyryl-CoA

**Fig. 1:**
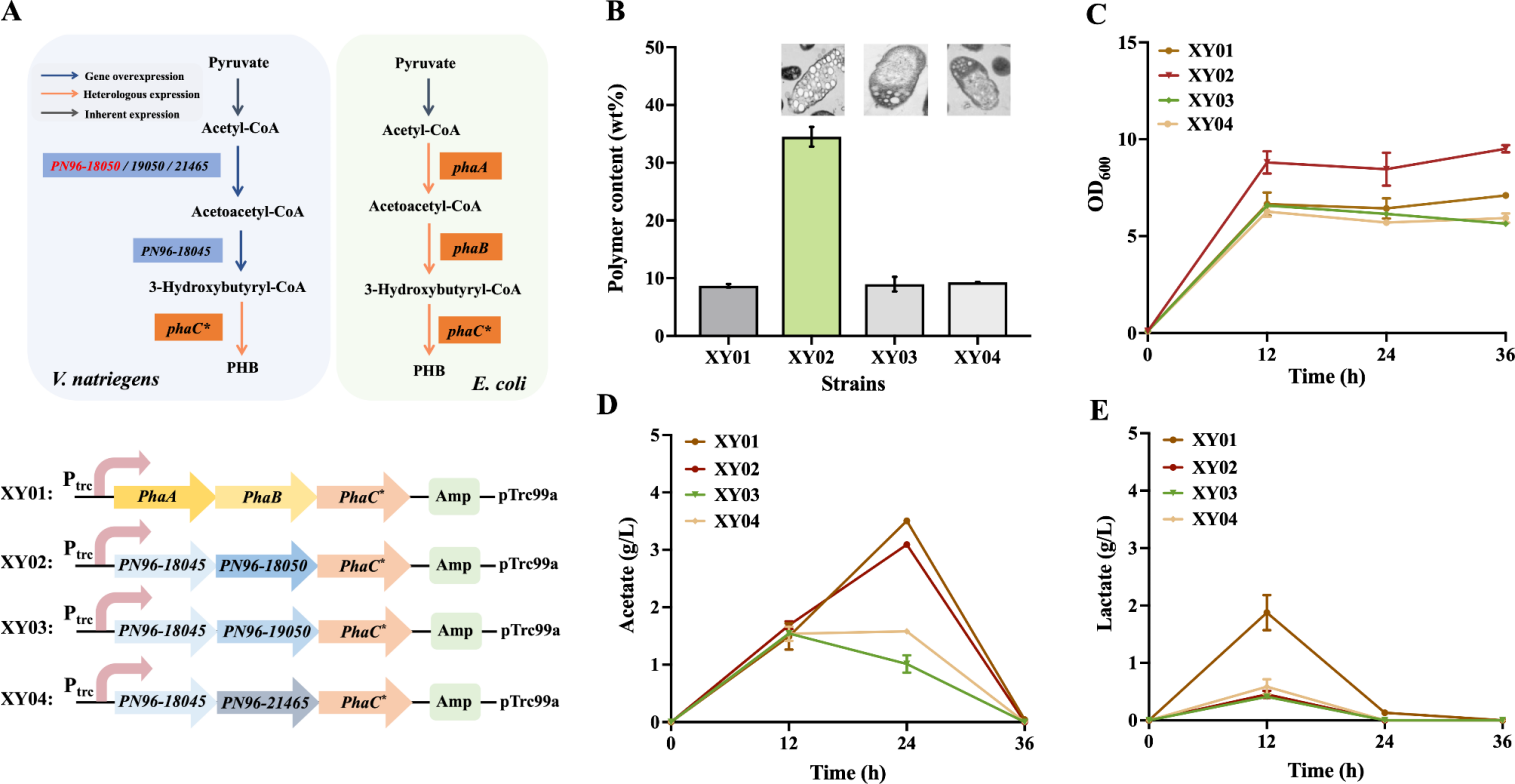
Identification of phaA enzyme in *V. natriegens.* (**A**) Metabolic pathway diagram of PHB production in *V. natriegens* and *E. coli*. phaA, β-ketothiolase; phaB, NADPH-dependent acetoacetyl-CoA reductase; phaC*, polyhydroxyalkanoate synthase mutant from *P. fluorescens*. (**B**) The production of PHB by XY01, XY02, XY03, and XY04. (**C**) The OD_600_ curves of mutant strains. (**D**) Profiles of acetate . (**E**) Profiles of lactate.

Currently, the synthesis of P(3HB-co-LA) has been achieved by modifying *E. coli* in our lab, which is a copolymer formed by the polymerization of two precursors lactyl-CoA and 3HB-CoA under the catalysis of a mutant PHA synthetase (phaC*) (Wu et al. 2021). 3HB-CoA is synthesized from acetyl-CoA via the enzymes *phaA* and NADPH-dependent acetoacetyl-CoA reductase *phaB* (Taguchi et al. 2008). Since *V. natriegens* naturally produces PHB, providing a complete pathway for PHB synthesis. Leveraging this pathway, the initial steps of PHB synthesis in *V. natriegens* can be utilized to produce the required precursor, 3HB-CoA (Dalia et al. 2017). Based on this, we identified the endogenous genes of PHB synthesis in *V. natriegens* firstly. According to the KEGG website (Ogata et al. 1999), there are three genes for acetyl-CoA acetyltransferase in *V. natriegens*, namely *PN96-18050*, *PN96-19050*, and *PN96-21465* (Fig. 1A). Here, exogenous genes of PHB synthesis commonly used in *E. coli* were introduced for testing and comparison, aiming to identify the PHB production pathway with optimal efficiency in *V. natriegens*. To identify the key enzymes of the PHB biosynthesis in *V. natriegens*, the expression plasmids of pTrc99a-phaABC*, pTrc99a-18050-18045-phaC*, pTrc99a-19050-18045-phaC* and pTrc99a-21465-18045-phaC* were introduced into strain *V. natriegens*, and named as XY01, XY02, XY03 and XY04, respectively (Fig. 1A). Strains XY02 showed a significant increase in PHB production compared to the other strains (34.5 wt%), suggesting that *PN96-18050* gene plays a critical role in precursor supplement of PHB production. The PHB content of XY01, XY03 and XY04 strains was 8.69 wt%, 8.96 wt% and 9.30 wt%, respectively (Fig. 1B). It seemed that overexpression of the other three acetyl-CoA acetyltransferases (*phaA*, *PN96-19050*, and *PN96-21465*) only led to a similar PHB accumulation. Meanwhile, it was observed that the titer of polymer components was also related to cell density, indicating that the increase of polymer titer was accompanied by the increase of OD_600_ (Fig. 1C). Moreover, we also detected the accumulations of lactate and acetate in the fermentation broth. At 24 h, XY01 exhibited the highest accumulation of acetate, reaching 3.5 g/L, indicative of pronounced metabolic overflow (Fig. 1D) (Majewski and Domach 1990). The acetate accumulation varied among strains: 3.1 g/L (XY02), 2.4 g/L (WT), 1.6 g/L (XY04), and 1.0 g/L (XY03). This variation might be due to different performance of the overflow metabolism. The lactate accumulation in XY01 was 1.8 g/L, significantly higher than the lactate content (about 0.4 g/L) in the other three recombinant strains. The above results indicate that, compared to the introduction of exogenous expression modules, the overexpression of the endogenous gene *PN96-18050* is a more suitable and effective approach to promote PHB production in *V. natriegens*.

### *De novo* synthesis of P(3HB-co-LA) in *V. natriegens*

**Fig. 2:**
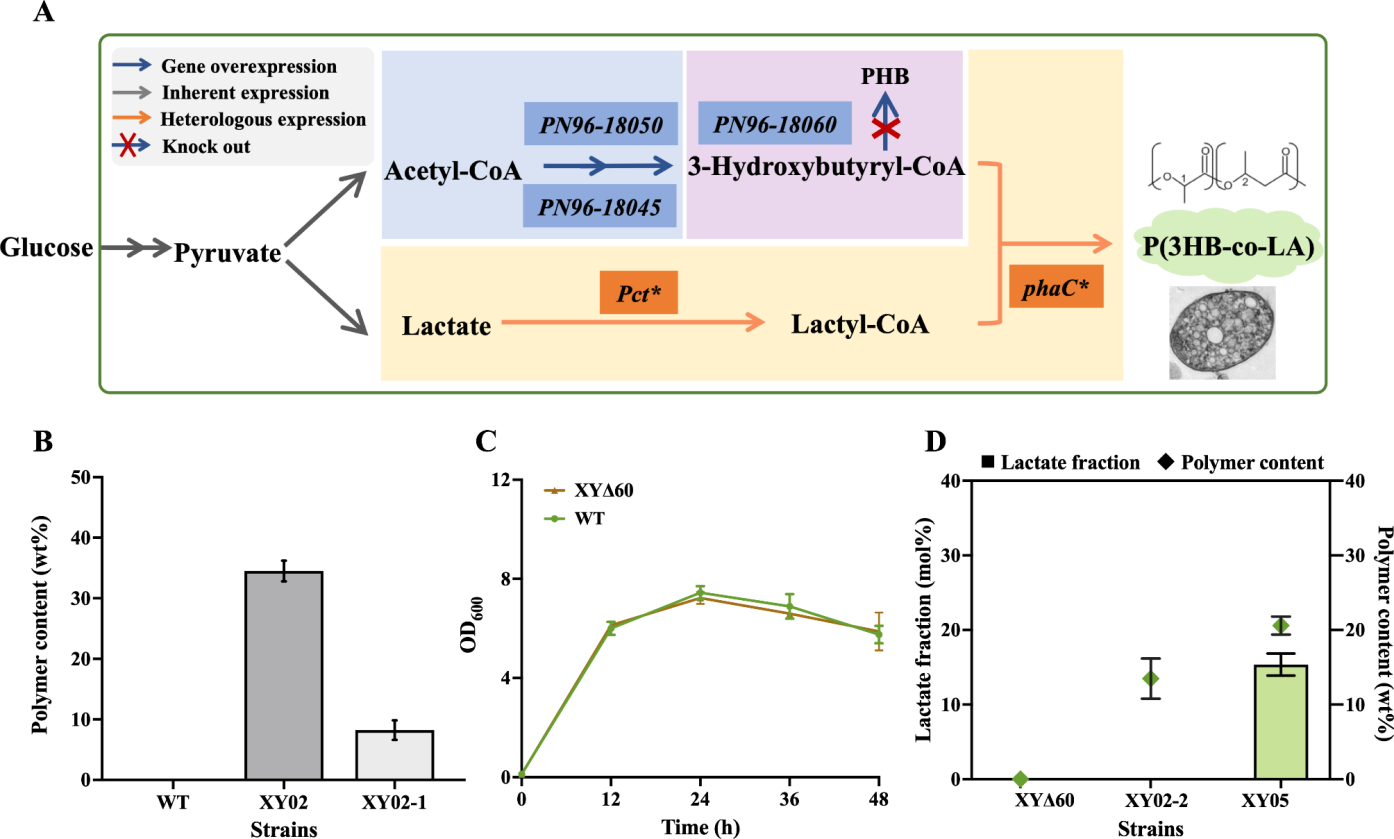
*De novo* production of P(3HB-co-LA) in *V. natriegens*. (**A**) Schematic diagram of biosynthetic pathways of P(3HB-co-LA) production in *V. natriegens.* (**B**) The PHB accumulation curve of engineered strains. (**C**) The growth curve of engineering strains. (**D**) Accumulation of P(3HB-co-LA) in the engineering strains

Based on the determination of the key gene for 3HB-CoA synthesis in *V. natriegens*, further introduction of lactyl-CoA biosynthesis pathway enables *de novo* synthesis of P(3HB-co-LA) (Fig. 2A). Since *V. natriegens* is a natural producer of PHB, its endogenous PHB synthase (PN96-18060) can polymerize 3HB-CoA to generate PHB, which may compete for the precursor in the synthesis of P(3HB-co-LA). PHB accumulation in *V. natriegens* also affect the detection of LA fraction. Therefore, we first characterized *V. natriegens* strains with enhanced 3HB-CoA synthesis modules. Strain XY02-1 was obtained by overexpressing the endogenous genes *PN96-18050* and *PN96-18045* in the wild-type strain. As shown in Fig. 2B, the wild-type strain did not accumulate PHB, which may be due to the fact that PHB accumulates more easily under nutrient-limited conditions. Although PHB accumulation was not detected in wild-type *V. natrigens*, the accumulation of PHB was observed in *V. natriegens* after enhancing the supply of endogenous 3HB-CoA. Consequently, it is necessary to knock out the endogenous PHB synthase gene *PN96-18060* in *V. natriegens*. *PN96-18060* knockout did not affected the growth compared with the wild-type VNT (Fig. 2C). The PHB synthesis pathway in XY02 was blocked by knockout of *PN96-18060* to obtain XY02-2, and mutant pct* gene isolated from *C. propionicum* was also introduced in XY02-2 to obtain strain XY05. As depicted in Fig. 2D, by addition of pct* gene, XY05 was able to synthesize P(3HB-co-LA) containing lactate components compared with strain XY02-2. The engineered strain XY05 achieved *de novo* synthesis of P(3HB-co-LA), with a copolymer content of 20.6 wt%, where the LA fraction was 15.4 mol%.

### Effect of *dldh* overexpression on the lactate fraction of P(3HB-co-LA)

**Fig. 3:**
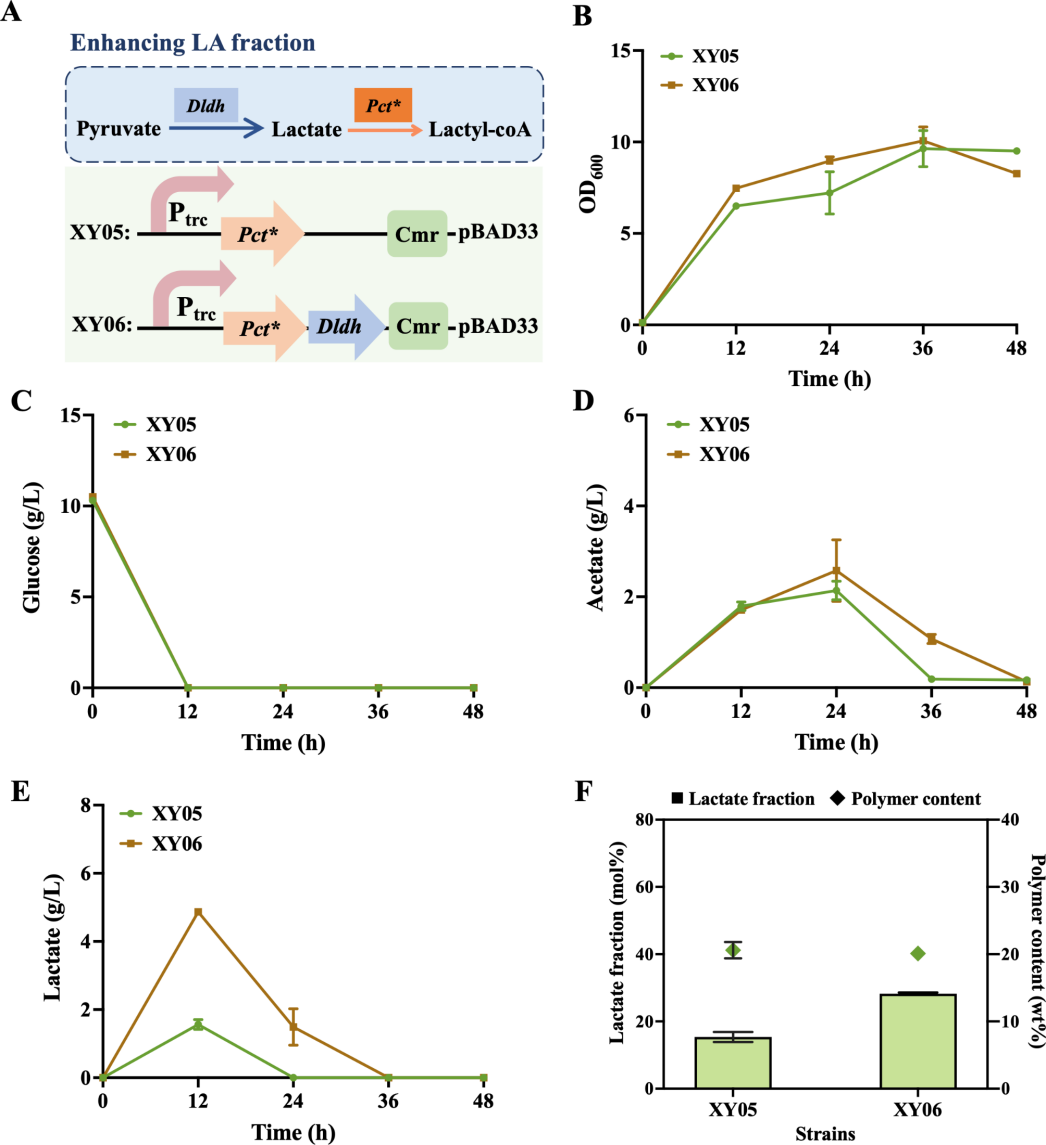
Increase the lactate component in P(3HB-co-LA). (**A**) The genetic manipulation of engineered strains. (**B**) The OD_600_, (**C**) glucose consumption, (**D**) lactate content, and (**E**) acetate content curves of mutant strains. (**F**) Accumulation of polymer content and lactate fraction levels by different strains

The LA fraction in P(3HB-co-LA) significantly influences its properties, wherein higher LA fraction correlates with increased material transparency and improved mechanical performance. The transparency of the copolymer increases with the LA fraction, while the decreasing PHB content reduces brittleness, enhancing toughness and elongation. At an LA fraction of 33.0 mol%, the elongation rate improves 4-fold, approaching that of conventional polyethylene plastics (Daisuke et al., 2017). In previous studies, several strategies have been employed to regulate and enhance the LA fraction in P(3HB-co-LA) (Yamada et al. 2011). Notably, the *dldh* gene, encoding D-lactate dehydrogenase, facilitates the production of D-lactate from pyruvate, thereby supplying the requisite precursor for Lactyl-CoA synthesis. Hence, to investigate the effect of *dldh* overexpression on the LA fraction, XY06 was constructed based on the engineered strain XY05, which possesses the P(3HB-co-LA) biosynthesis pathway (Fig. 3A). Upon observing that the overexpression of *dldh* did not affect the growth and glucose consumption (Fig. 3B), however, the significant changes were noted in the lactate accumulation between strains XY05 and XY06 during fermentation. Specifically, the lactate content in the supernatant of XY06 medium was twice that of XY05 at 12 h (Fig. 3E). The LA fraction in the polymers of XY06 reached to 28.2 mol%, was almost twice that of XY05 (15.3 mol%) (Fig. 3F). Both of the strains reached their highest acetate content at 24 h, however, the consumption rate of XY06 was lower than that of XY05 due to the higher concentration of lactate (Fig. 3D and E). It seemed that the engineered strains prefer to use lactate than acetate. The above results indicate that overexpression of *dldh* in *V. natriegens* effectively catalyzes the production of lactate, providing more precursors for lactyl-CoA, thereby increasing the content of lactate in the polymer.

### P(3HB-co-LA) production using different carbon sources

**Fig. 4:**
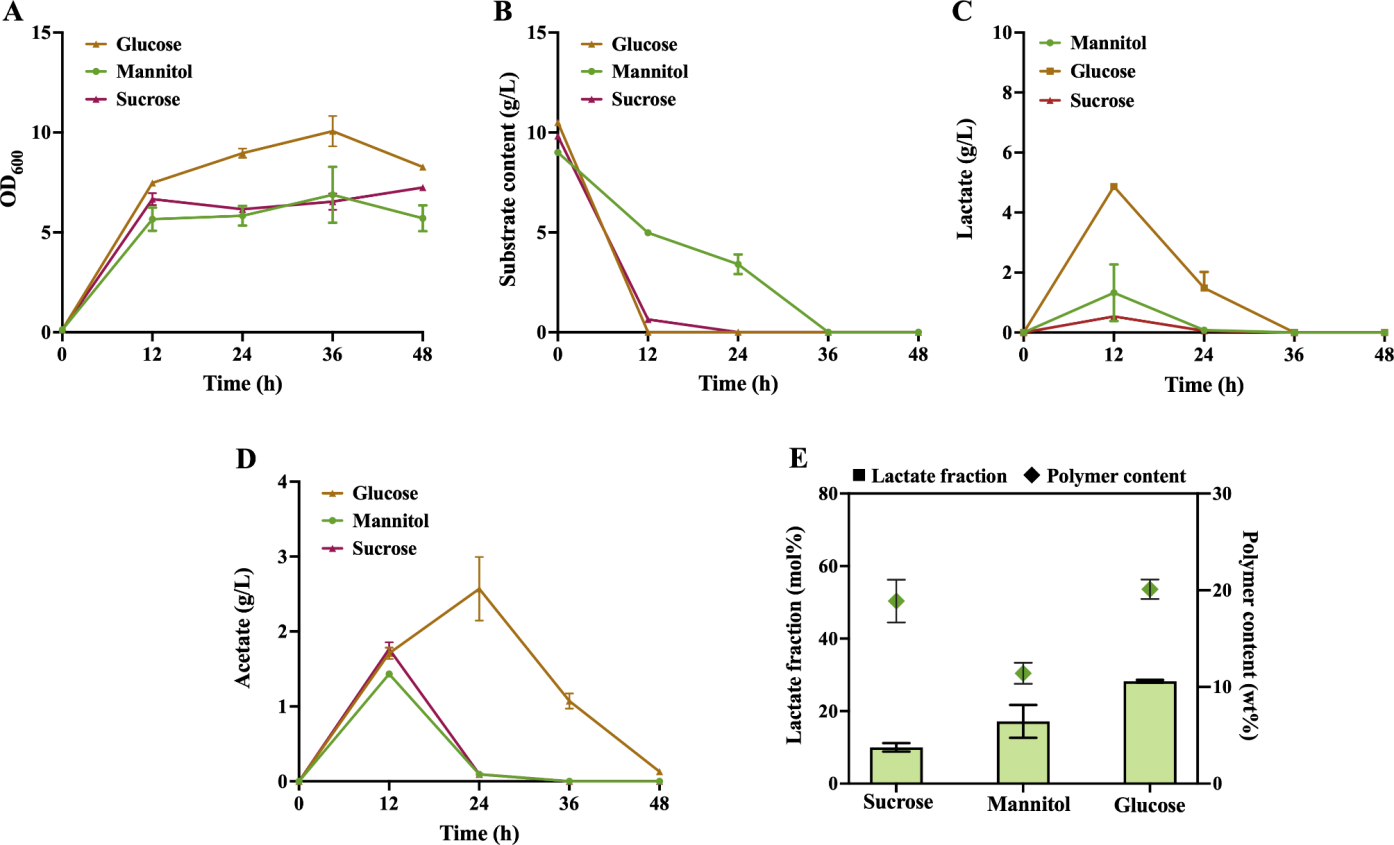
Explore the substrate utilization capacity. Characterization curves of cell density (**A**), substrate content (**B**), lactate content (**C**), acetate content (**D**), polymer content (**E**), and lactate fraction under different carbon source culture conditions by XY06

Considering the wide substrate adaptability of *V. natriegens*, we explored its potential to produce P(3HB-co-LA) in various substrates. Sucrose, as a cheap and readily available carbon source, has potential application prospects. *V. natriegens* can directly use sucrose as a carbon source (Lee et al. 2024). In addition, marine macroalgae have been identified as a promising renewable resource in the field of industrial fermentation. Mannitol, as one of the highest content sugars in brown algae, can be easily obtained (Gnaim et al. 2022). Therefore, we chose sucrose and mannitol as the sole carbon sources for testing. It was observed that the growth of *V. natriegens* on glucose was better than those of sucrose and mannitol (Fig. 4A), and the substrate uptake capacity of mannitol was lower than the other two carbon sources (Fig. 4B). When using sucrose as a carbon source, XY06 can produce 18.9 wt% polymer, the polymer yield is similar to glucose. However, when sucrose is used as the substrate, the lactate fraction is only about 35.0 mol% of that in glucose production. Compared the concentration of accumulated lactate with that of glucose, we speculated that this may be due to insufficient accumulation of the intracellular lactate when sucrose is used as a substrate. When using mannitol as the substrate, XY06 can produce 20.1 wt% P(3HB-co-LA), with a LA fraction of 17.1 mol%. Compared with glucose fermentation, the yield and performance of the LA fraction have decreased (Fig. 4C). As shown in Fig. 4E, glucose has been proven to be the best substrate with the highest LA fraction. When different substrates were used, the accumulation of in XY06 were different. The highest accumulation of acetate occurred when glucose was used as substrate, reaching 2.57 g/L in 24 h. When sucrose and mannitol were used as substrates, the highest content at 12 h was 1.77 g/L and 1.43 g/L, separately (Fig. 4D). The content of acetate was almost exhausted at 24 h, and the metabolic overflow was significantly lower than that of glucose. The possible reason is that glucose is a rapidly available carbon source, which is easy to produce obvious acetate overflow, so it is necessary to explore and use other carbon sources to produce P(3HB-co-LA). Therefore, the substrate utilization pathway can be further strengthened in the future to increase the accumulation of P(3HB-co-LA) and LA fraction.

## Conclusion

P(3HB-co-LA) has a widespread application and substantial market demand due to outstanding toughness and transparency. Although the biosynthesis of P(3HB-co-LA) has been achieved and progress has been made in optimizing synthesis pathways and fermentation processes, developing microbial cell factories that meet industrial-scale production requirements remains challenging. This study explores the use of *V. natriegens*, considered a “next-generation synthetic biology chassis,” due to its broad substrate utilization and rapid growth. In this work, the biosynthesis of P(3HB-co-LA) in *V. natriegens* was successfully established for the first time. By identifying the endogenous rate-limiting gene, knockout the key enzyme of endogenous PHB biosynthesis and overexpression of the P(3HB-co-LA) biosynthesis pathway, about 20.6 wt% of P(3HB-co-LA) was successfully produced, and the LA fraction was increased to 28.3 mol%. Furthermore, mannitol and sucrose were used as substrates for P(3HB-co-LA) production. In summary, this work represents the first successful production of P(3HB-co-LA) in *V. natriegens*, offering the significant potential of engineered *V. natriegens* strains for PHAs production using various carbon sources.

## Electronic supplementary material

Below is the link to the electronic supplementary material.

Supplementary Material 1

Supplementary Material 2

## Abbreviations

P(3HB-co-LA): Poly(3-hydroxybutyrate-co-lactate)
V. natriegens: Vibrio natriegens
LA: Lactate
PHA: Polyhydroxyalkanoates
PLA: Polylactic acid
PHB: Polyhydroxybutyrate
3HB-CoA: 3-hydroxybutyryl-CoA
phaC*: mutant PHA synthetase
phaB: NADPH-dependent acetoacetyl-CoA reductase
phaA: acetyl-CoA acetyltransferases
Dldh: D-lactate dehydrogenase
Pct*: Mutant propionyl-CoA transferase
