# Correction: Engineering of fast-growing Vibrio natriegens for biosynthesis of poly(3-hydroxybutyrate-co-lactate)

**DOI:** 10.1186/s40643-024-00811-2

**Published:** 2024-10-14

**Authors:** Xinye Sun, Yanzhe Shang, Binghao Zhang, Pengye Guo, Yuanchan Luo, Hui Wu

**Affiliations:** 1 State Key Laboratory of Bioreactor Engineering, Shanghai Frontiers Science Center of Optogenetic Techniques for Cell Metabolism, School of Biotechnology, East China University of Science and Technology, 130 Meilong Road, Shanghai, 200237 China; 2 MOE Key Laboratory of Bio-Intelligent Manufacturing, School of Bioengineering, Dalian University of Technology, Dalian, China; 3 Shanghai Collaborative Innovation Center for Biomanufacturing Technology, 130 Meilong Road, Shanghai, 200237 China; 4 Key Laboratory of Bio-based Material Engineering of China, National Light Industry Council, 130 Meilong Road, Shanghai, 200237 China

**Correction: Bioresources and Bioprocessing (2024) 11:86**

10.1186/s40643-024-00801-4

The wrong Supplementary file was originally published with this article; it has now been replaced with the correct file. There is only one “Supplementary Material”.

The following references have been corrected in the Original article.

Daisuke I, Kenji T (2017) Ken’ichiro, Matsumoto, Toshihiko, Ooi, Takaaki, Hikima Effect of monomeric composition on the thermal, mechanical and crystalline properties of poly[(R)-lactate-co^®^3-hydroxybutyrate]. 10.1016/j.polymer.2017.06.039.

Gao D, Liu T, Gao J, Xu J, Gou Y, Pan Y, Li D, Ye C, Pan R, Huang L, Xu Z, Lian J (2022) De Novo Biosynthesis of Vindoline and Catharanthine in Saccharomyces cerevisiae. BioDesign Res. 10.34133/bdr.0002

Hoff J, Daniel B, Stukenberg D, Thuronyi BW, Waldminghaus T, Fritz G (2020) Vibrio natriegens: an ultrafast-growing marine bacterium as emerging synthetic biology chassis. Environ Microbiol. 10.1111/1462-2920.15128.

Lu H, Yuan G, Strauss SH, Tschaplinski TJ, Tuskan GA, Chen J-G, Yang X (2020) Reconfiguring plant metabolism for biodegradable plastic production. Biodes Res 2020. 10.34133/2020/9078303.

McAdam B, Brennan Fournet M, McDonald P, Mojicevic M (2020) Production of polyhydroxybutyrate (PHB) and factors impacting its chemical and mechanical characteristics. Polymers 12(12). 10.3390/polym12122908.

Shi Z, Liu P, Liao X, Mao Z, Zhang J, Wang Q, Sun J, Ma H, Ma Y (2022) Data-driven synthetic cell factories development for Industrial Biomanufacturing. 10.34133/2022/9898461.

BioDesign Research 2022(9898461.

Thompson MG, Moore WM, Hummel NFC, Pearson AN, Barnum CR, Scheller HV, Shih PM (2020) Agrobacterium tumefaciens: A bacterium primed for synthetic biology. Biodes Res 2020 (10.34133/2020/8189219.

The correct ones are as followed:

Ishii D, Takisawa K, Matsumoto K, Ooi T, Hikima T, Takata M, Taguchi S, Iwata T (2017) Effect of monomeric composition on the thermal, mechanical and crystalline properties of poly[(R)(R)-3-hydroxybutyrate]. Polymer 122:169–173. 10.1016/j.polymer.2017.06.039.

Gao D, Liu T, Gao J, Xu J, Gou Y, Pan Y, Li D, Ye C, Pan R, Huang L, Xu Z, Lian J (2022) De Novo Biosynthesis of Vindoline and Catharanthine in Saccharomyces cerevisiae. BioDesign Res. 2022. 0002. 10.34133/bdr.0002.

Hoff J, Daniel B, Stukenberg D, Thuronyi BW, Waldminghaus T, Fritz G (2020) Vibrio natriegens: an ultrafast-growing marine bacterium as emerging synthetic biology chassis. Environ Microbiol. 22(10):4394–4408. 10.1111/1462-2920.15128.

Lu H, Yuan G, Strauss SH, Tschaplinski TJ, Tuskan GA, Chen J-G, Yang X (2020) Reconfiguring plant metabolism for biodegradable plastic production. Biodes Res. 2020. 9,078,303. 10.34133/2020/9078303.

McAdam B, Brennan Fournet M, McDonald P, Mojicevic M (2020) Production of polyhydroxybutyrate (PHB) and factors impacting its chemical and mechanical characteristics. Polymers 12(12):2908. 10.3390/polym12122908.

Shi Z, Liu P, Liao X, Mao Z, Zhang J, Wang Q, Sun J, Ma H, Ma Y (2022) Data-driven synthetic cell factories development for Industrial Biomanufacturing. BioDesign Res. 2022. 9,898,461. 10.34133/2022/9898461.

Thompson MG, Moore WM, Hummel NFC, Pearson AN, Barnum CR, Scheller HV, Shih PM (2020) Agrobacterium tumefaciens: A bacterium primed for synthetic biology. Biodes Res. 2020. 8,189,219. 10.34133/2020/8189219.

