# Supplementary figures and images for: Engineering of fast-growing Vibrio natriegens for biosynthesis of poly(3-hydroxybutyrate-co-lactate)
Source: Bioresour Bioprocess. 2024 Sep 9;11(1):86. doi: 10.1186/s40643-024-00801-4 (PMC11383894; doi:10.1186/s40643-024-00801-4)

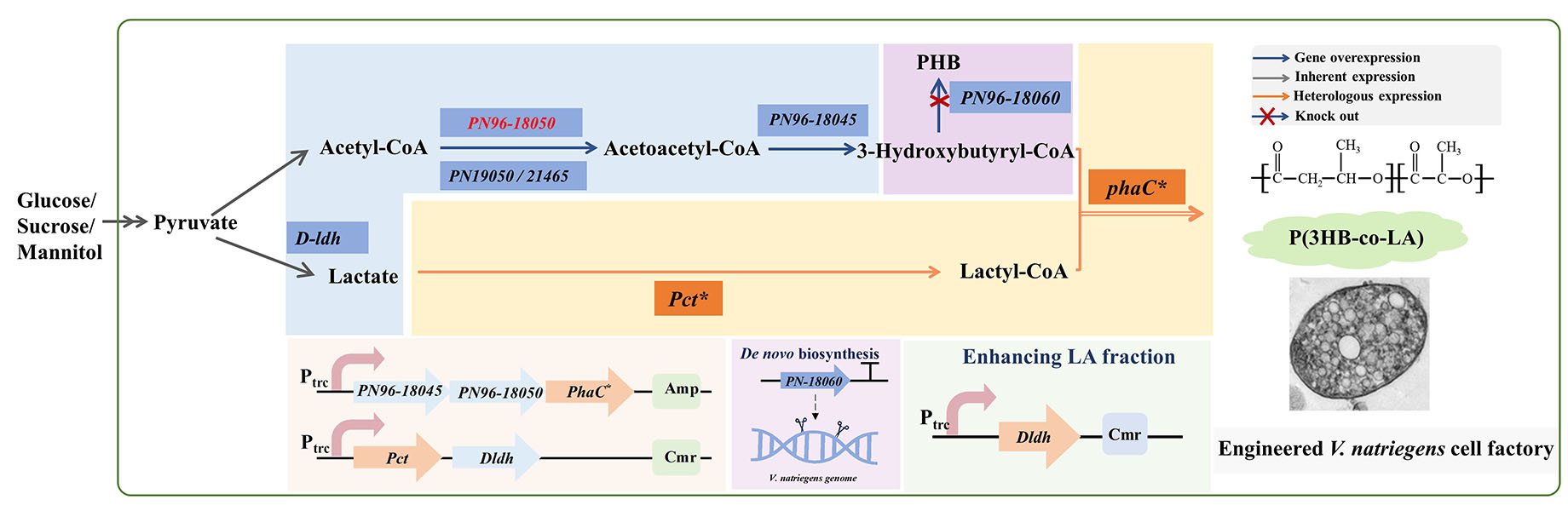

Supplement: Supplementary file 1 — Supplementary Material 1 [file 40643_2024_801_MOESM1_ESM.jpg]
