# Supplementary material for: Engineering of fast-growing Vibrio natriegens for biosynthesis of poly(3-hydroxybutyrate-co-lactate)
Source: Bioresour Bioprocess. 2024 Sep 9;11(1):86. doi: 10.1186/s40643-024-00801-4 (PMC11383894; doi:10.1186/s40643-024-00801-4)
Supplement: Supplementary file 2 — Supplementary Material 2 [file 40643_2024_801_MOESM2_ESM.docx]

**Supplementary Information**

**Engineering of fast-growing *Vibrio natriegens* for biosynthesis of poly(3-hydroxybutyrate-co-lactate)**

**Table S1.** Strains and plasmids used in this study

| Strains/plasmids | Characteristic | Reference |
| --- | --- | --- |
| pTrc99a | Cloning vector, *trc* promotor, Ap^R^ | Laboratory collection |
| pBAD33 | Cloning vector, *araC* promotor, Chl^R^ | Laboratory collection |
| pTrc99a-phaABC* | pTrc99a containing the codon-optimized *phaC** gene from *P. fluorescens* strain 2P24 (E130D, S325T, Q481K), *phaA* and *phaB* gene from *Ralstonia eutropha* | This study |
| pBAD33-pct* | pBAD33 containing the codon-optimized *pct* mutant gene from *C. propionicum* DSM 1682 | This study |
| pTrc99a-18045-18050-phaC* | pTrc99a containing the codon-optimized *phaC**, *PN96-18050* and *PN96-18045* gene from *V. natriegens* | This study |
| pTrc99a-19050-18045-phaC* | pTrc99a containing the codon-optimized *phaC**, *PN96-19050* and *PN96-18045* gene from *V. natriegens* | This study |
| pTrc99a-21465-18045-phaC* | pTrc99a containing the codon-optimized *phaC**, *PN96-21465* and *PN96-18045* gene from *V. natriegens* | This study |
| pTrc99a-18045-18050 | pTrc99a containing *PN96-21465* and *PN96-18045* gene from *V. natriegens* | This study |
| pBAD33-pctvn-dldh | pBAD33 containing the codon-optimized *pct* gene and *dldh* gene | This study |
| WT | Wild type *V. natriegens* | ATCC14048 |
| XY01 | WT containing pTrc99a-phaABC* | This study |
| XY02 | WT containing pTrc99a-18050-18045-phaC* | This study |
| XYΔ60 | WT ΔPN96-18060 | This study |
| XY02-1 | WT containing pTrc99a-18045-18050 | This study |
| XY02-2 | XYΔ60 containing pTrc99a-18050-18045-phaC* | This study |
| XY03 | WT containing pTrc99a-19050-18045-phaC* | This study |
| XY04 | WT containing pTrc99a-21465-18045-phaC* | This study |
| XY05 | XYΔ60 containing pTrc99a-18050-18045-phaC* and pBAD33-pct* | This study |
| XY06 | XYΔ60 containing pTrc99a-18050-18045-phaC* and pBAD33-pctvn-dldh | This study |
